# Supplementary figures and images for: The endoplasmic reticulum connects to the nucleus by constricted junctions that mature after mitosis
Source: EMBO Rep. 2024 Jun 14;25(7):18. doi: 10.1038/s44319-024-00175-w (PMC11239909; doi:10.1038/s44319-024-00175-w)

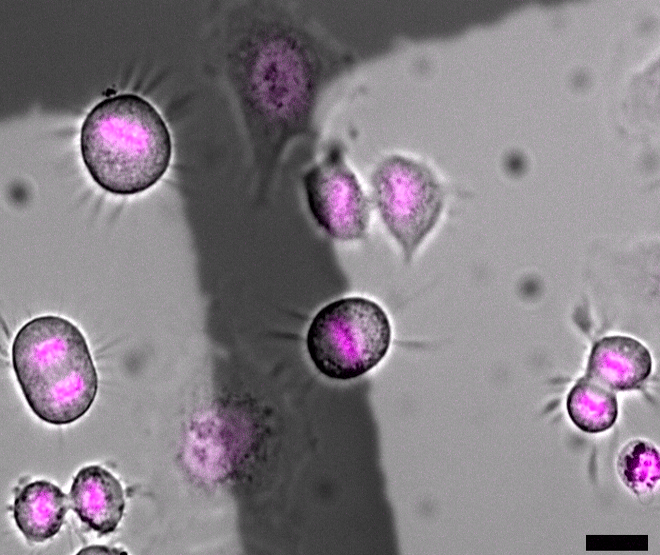

Supplement: Supplementary file 8 — Source data Fig. 1 [file 44319_2024_175_MOESM8_ESM.zip › Figure 1/1A/Fig1A_1.png]

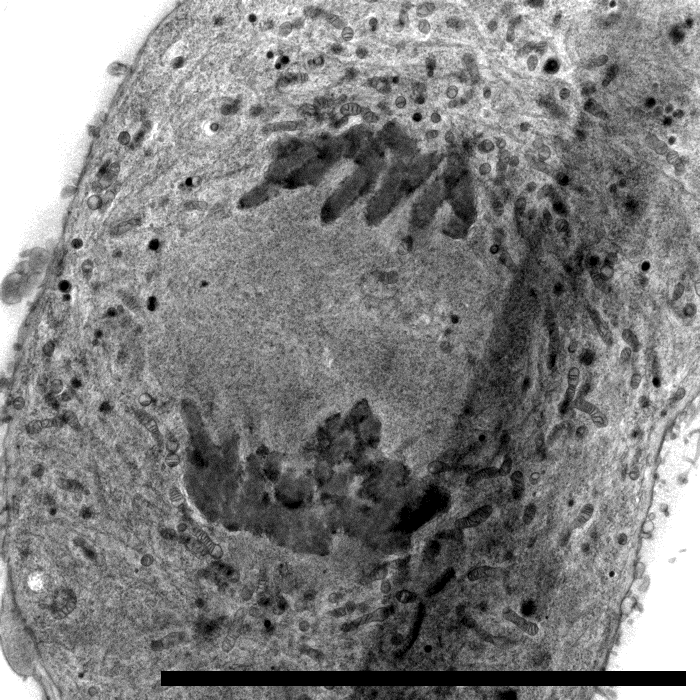

Supplement: Supplementary file 8 — Source data Fig. 1 [file 44319_2024_175_MOESM8_ESM.zip › Figure 1/1A/Fig1A_2.png]

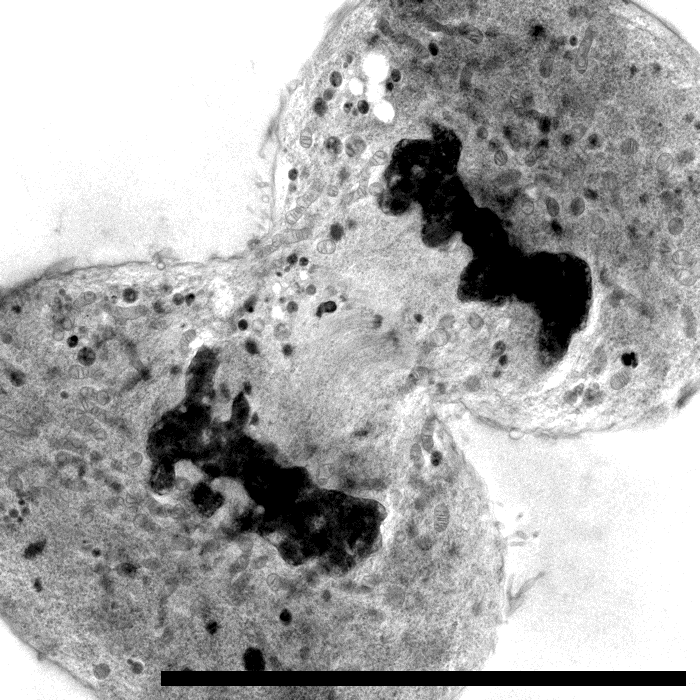

Supplement: Supplementary file 8 — Source data Fig. 1 [file 44319_2024_175_MOESM8_ESM.zip › Figure 1/1A/Fig1A_3.png]

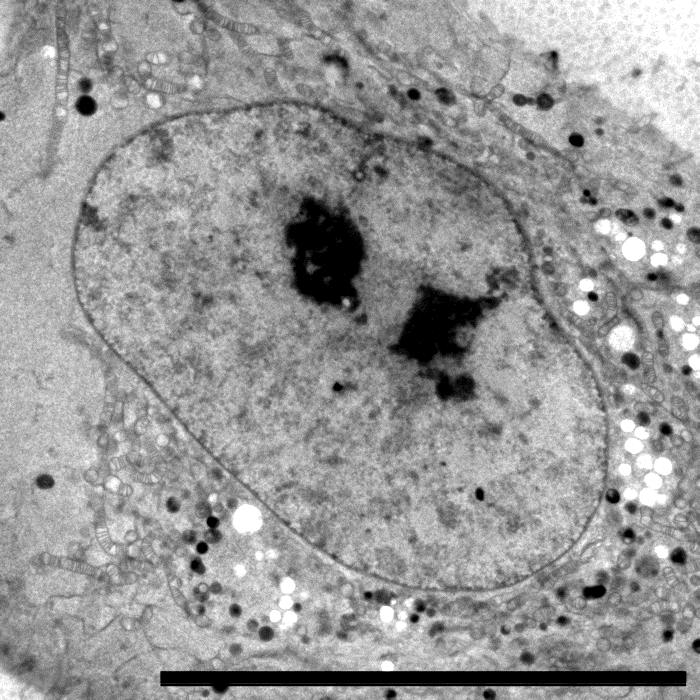

Supplement: Supplementary file 8 — Source data Fig. 1 [file 44319_2024_175_MOESM8_ESM.zip › Figure 1/1A/Fig1A_4.png]

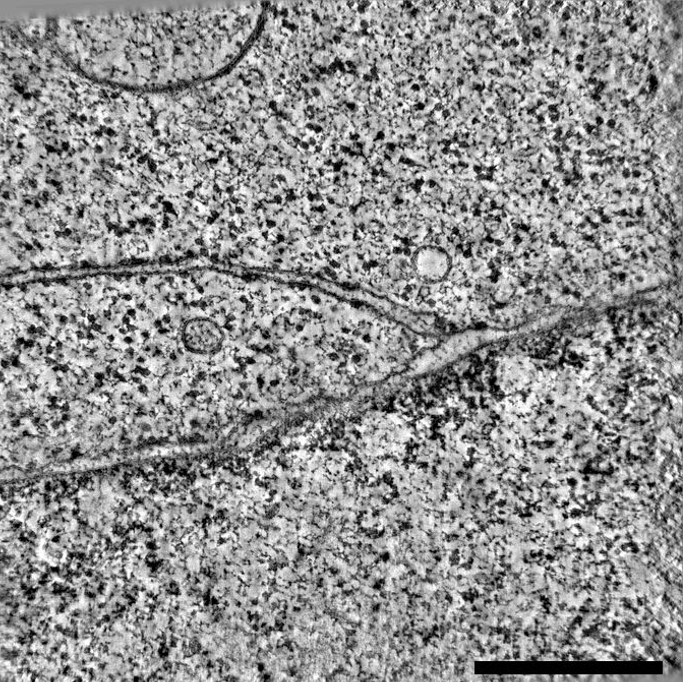

Supplement: Supplementary file 8 — Source data Fig. 1 [file 44319_2024_175_MOESM8_ESM.zip › Figure 1/1B/Fig1B_2.png]

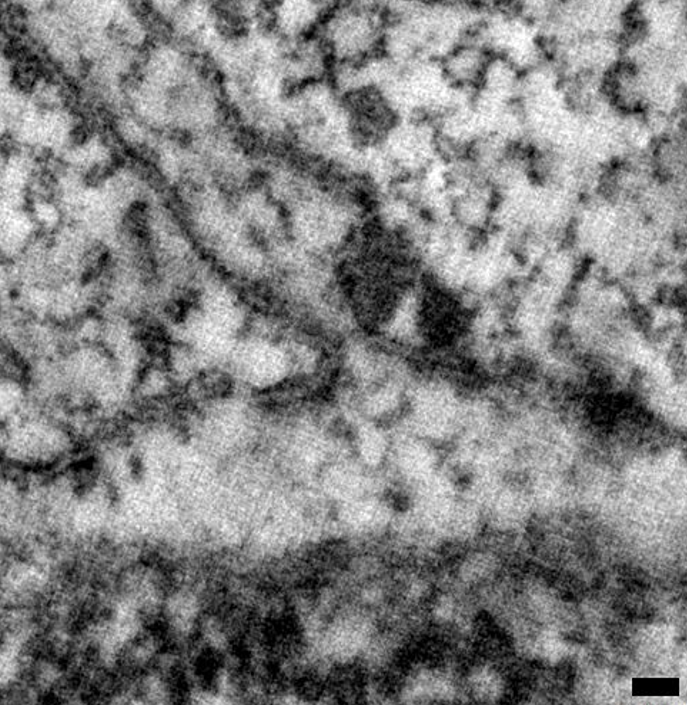

Supplement: Supplementary file 8 — Source data Fig. 1 [file 44319_2024_175_MOESM8_ESM.zip › Figure 1/1C/Fig1C.png]

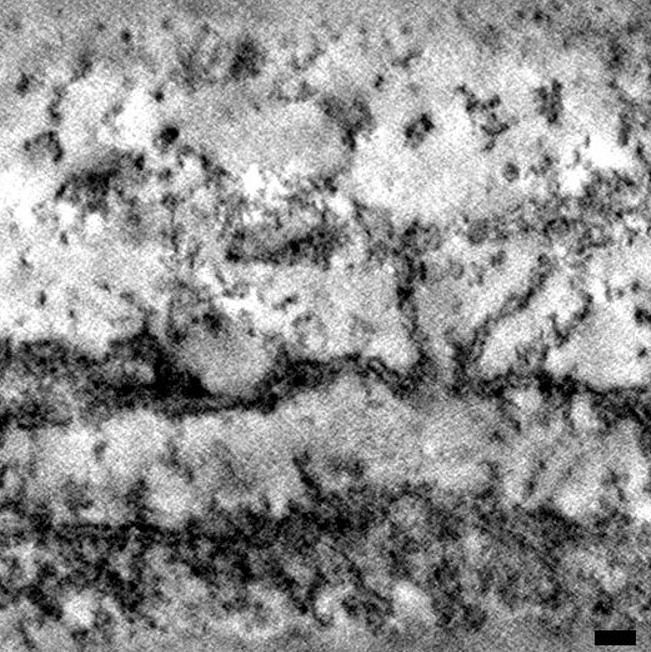

Supplement: Supplementary file 9 — Source data Fig. 2 [file 44319_2024_175_MOESM9_ESM.zip › Figure 2/2A/Fig2A_1.png]

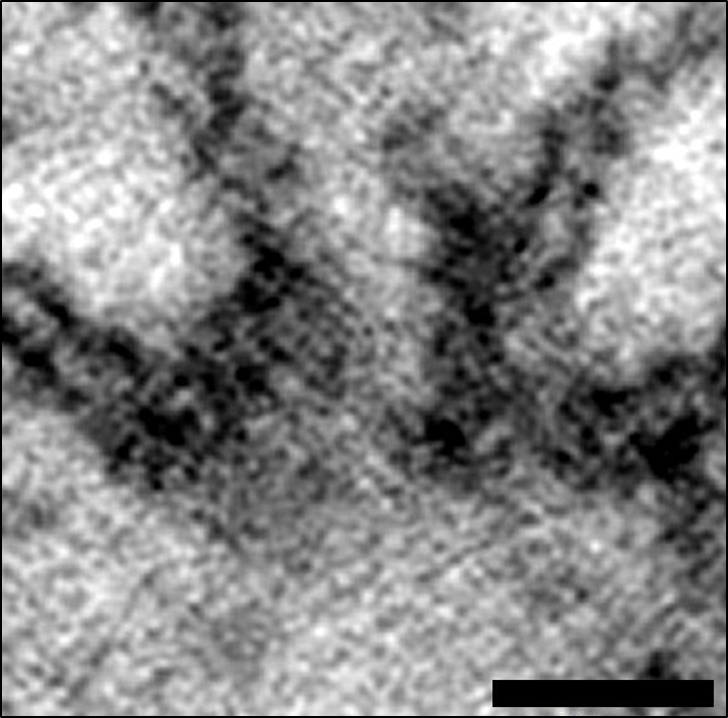

Supplement: Supplementary file 9 — Source data Fig. 2 [file 44319_2024_175_MOESM9_ESM.zip › Figure 2/2A/Fig2A_2.png]

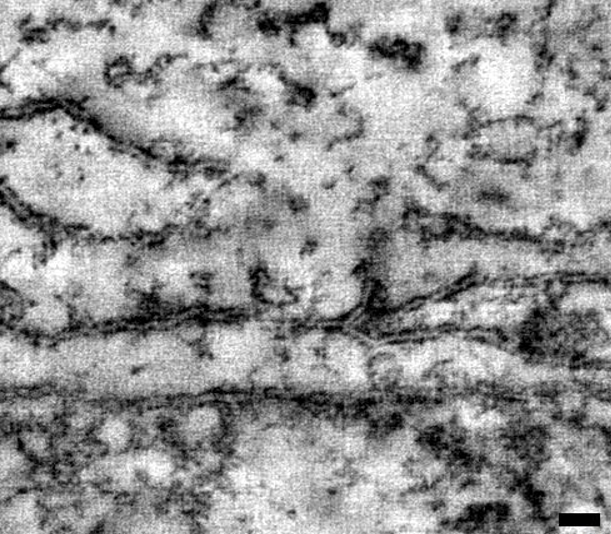

Supplement: Supplementary file 9 — Source data Fig. 2 [file 44319_2024_175_MOESM9_ESM.zip › Figure 2/2A/Fig2A_3.png]

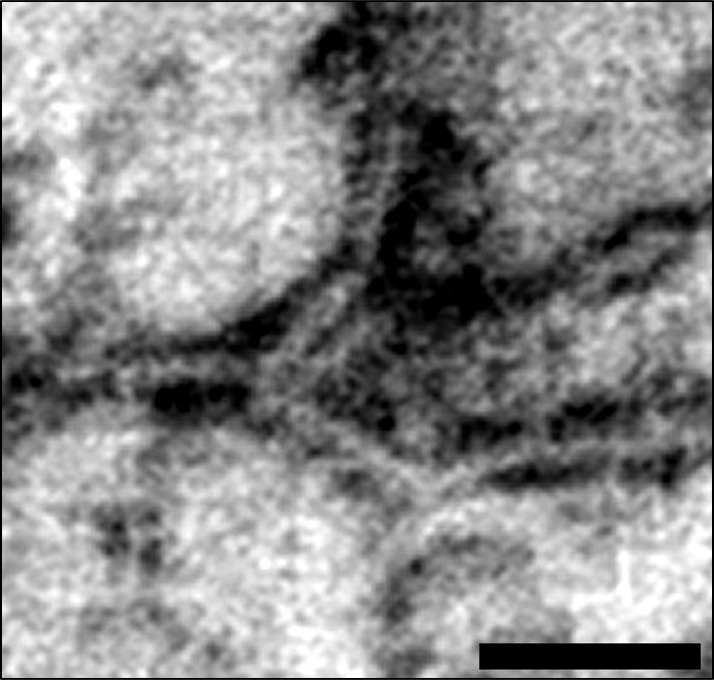

Supplement: Supplementary file 9 — Source data Fig. 2 [file 44319_2024_175_MOESM9_ESM.zip › Figure 2/2A/Fig2A_4.png]

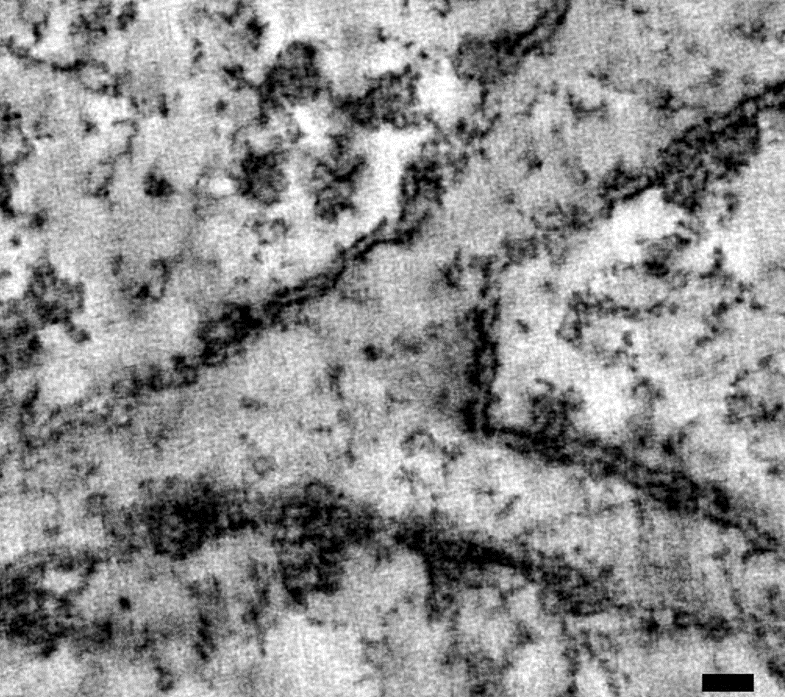

Supplement: Supplementary file 9 — Source data Fig. 2 [file 44319_2024_175_MOESM9_ESM.zip › Figure 2/2F/Fig2F_1.png]

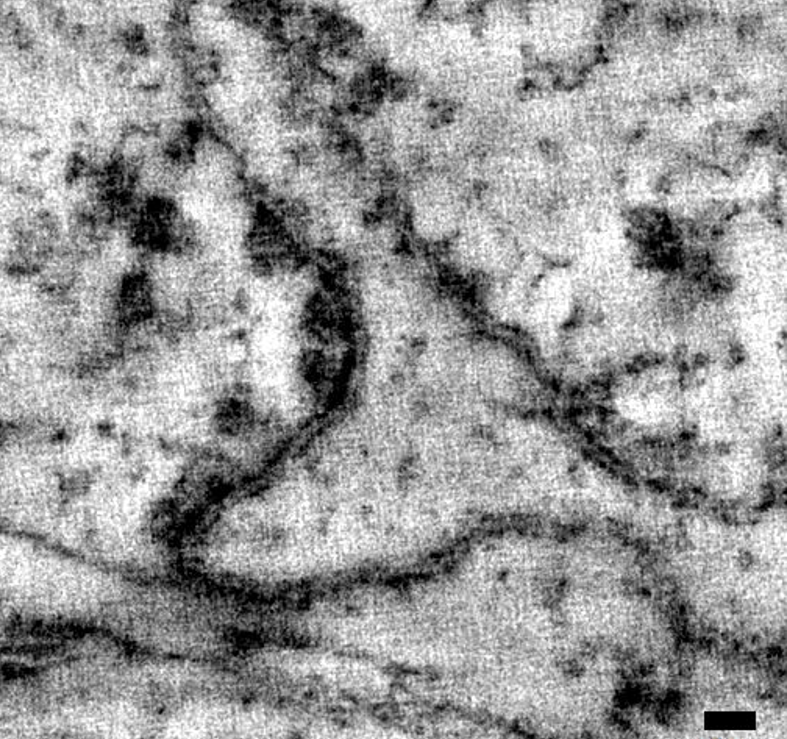

Supplement: Supplementary file 9 — Source data Fig. 2 [file 44319_2024_175_MOESM9_ESM.zip › Figure 2/2F/Fig2F_2.png]

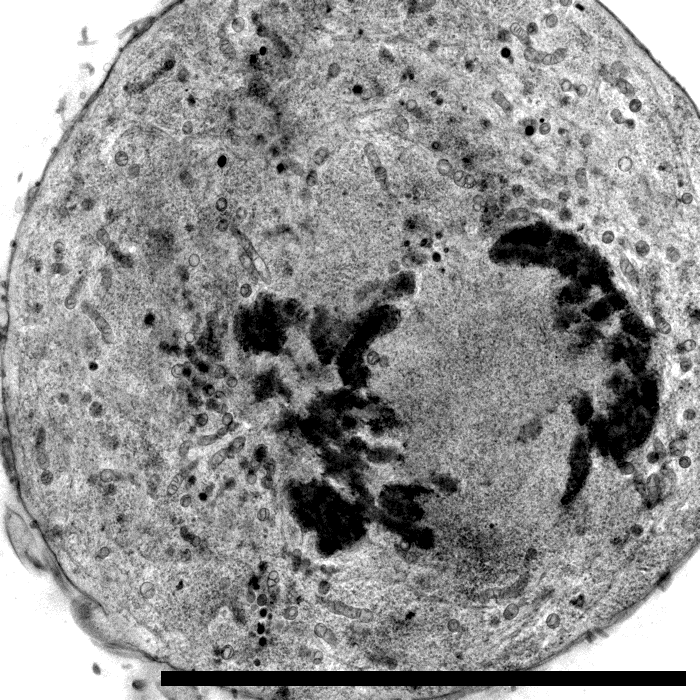

Supplement: Supplementary file 10 — Source data Fig. 3 [file 44319_2024_175_MOESM10_ESM.zip › Figure 3/3A/Fig3A_1.png]

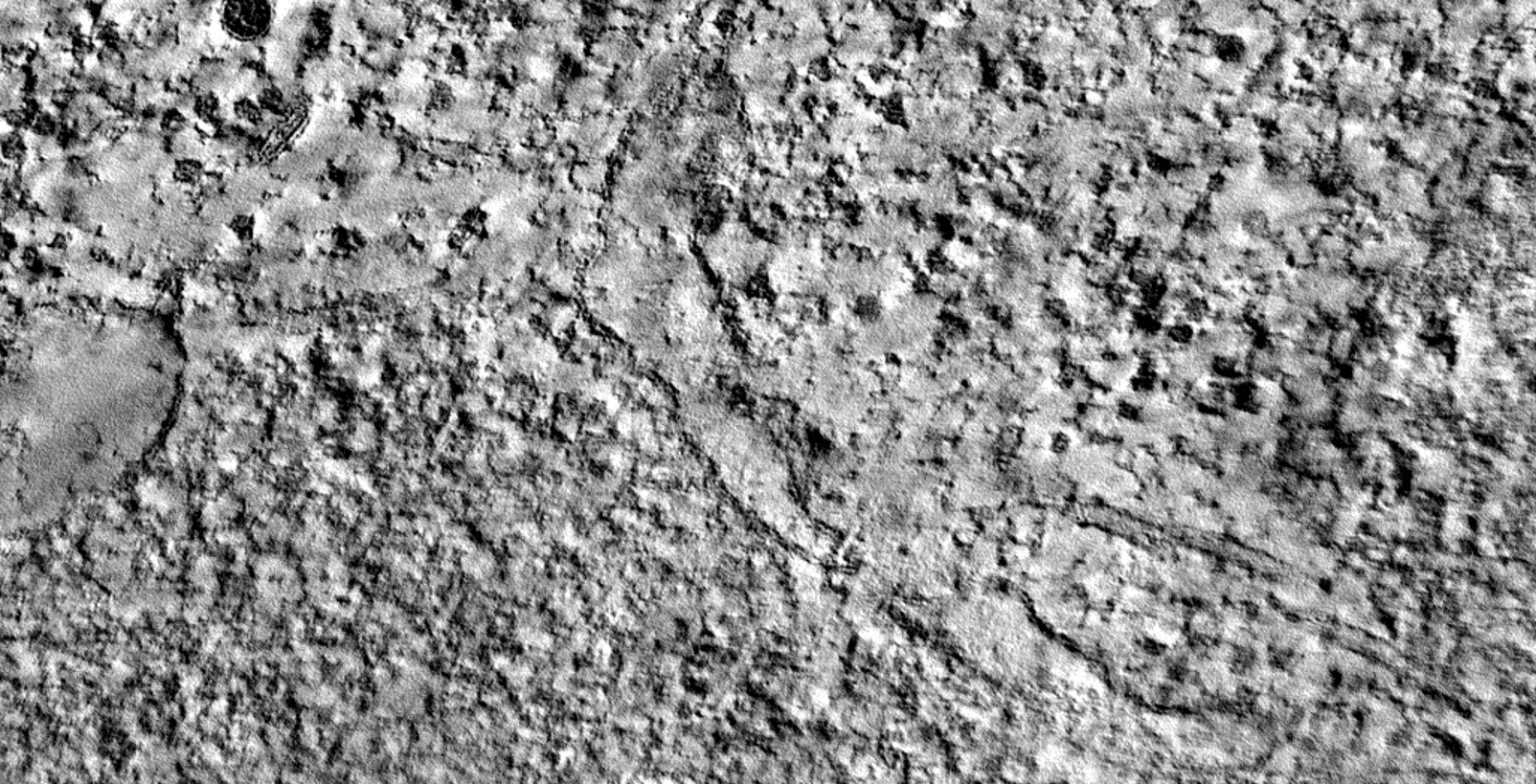

Supplement: Supplementary file 10 — Source data Fig. 3 [file 44319_2024_175_MOESM10_ESM.zip › Figure 3/3A/Fig3A_2.png]

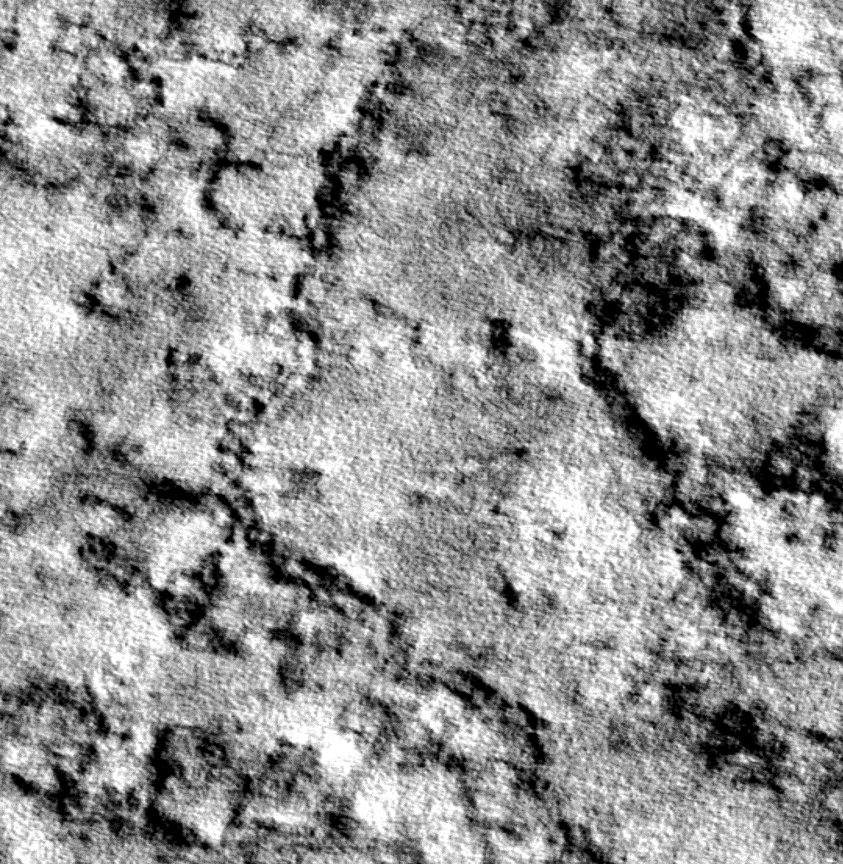

Supplement: Supplementary file 10 — Source data Fig. 3 [file 44319_2024_175_MOESM10_ESM.zip › Figure 3/3A/Fig3A_3.png]

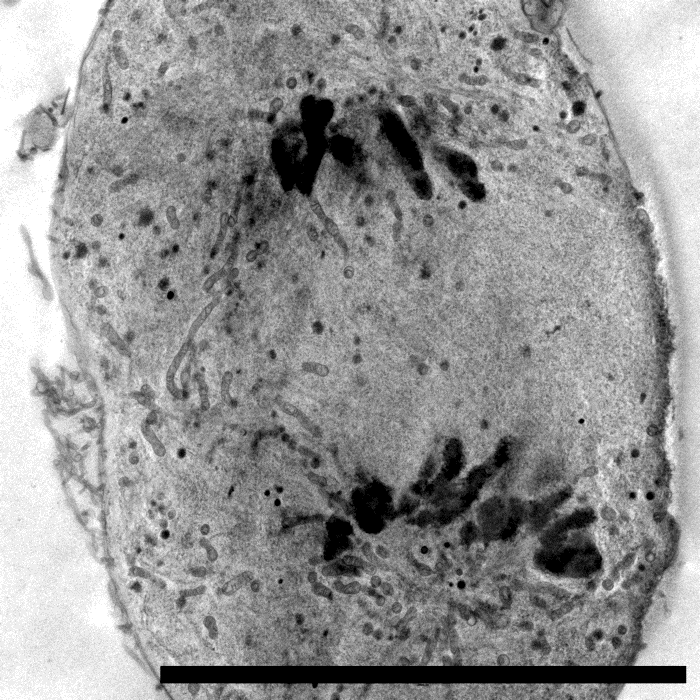

Supplement: Supplementary file 10 — Source data Fig. 3 [file 44319_2024_175_MOESM10_ESM.zip › Figure 3/3A/Fig3A_4.png]

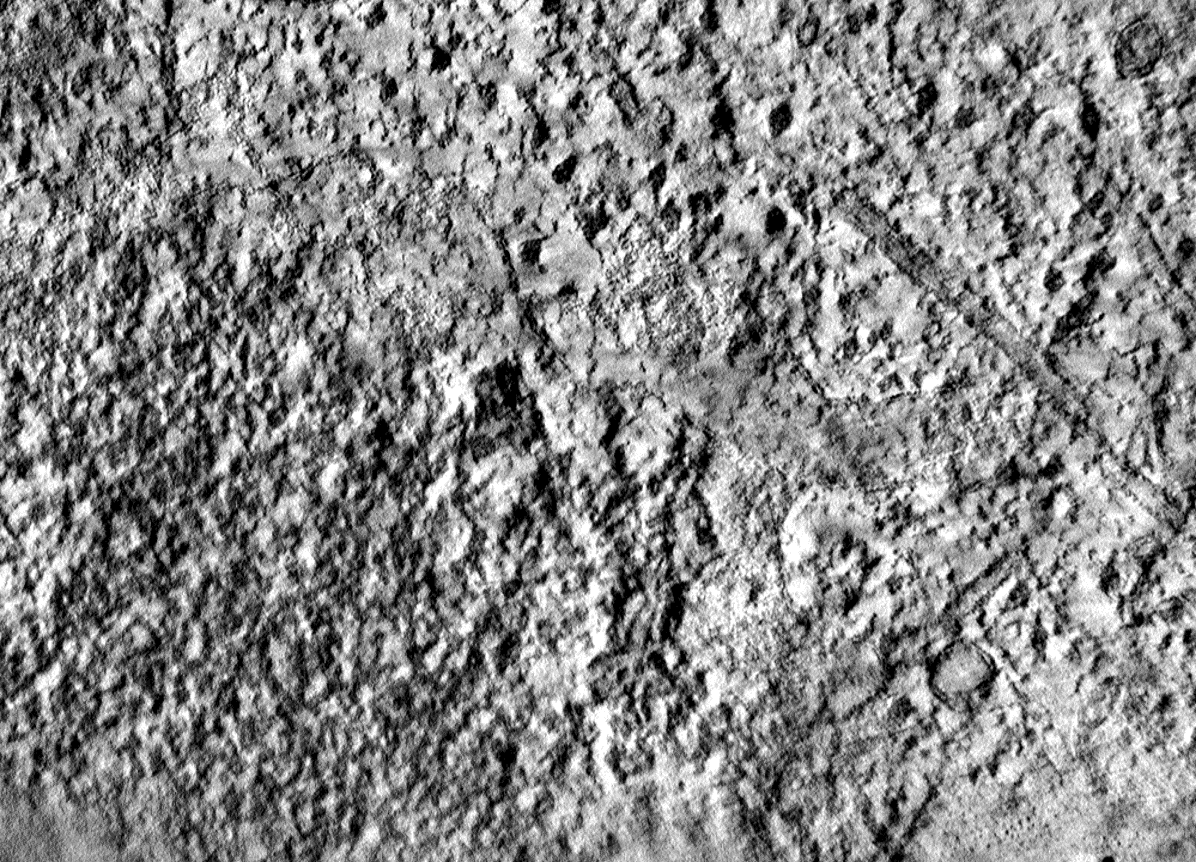

Supplement: Supplementary file 10 — Source data Fig. 3 [file 44319_2024_175_MOESM10_ESM.zip › Figure 3/3A/Fig3A_5.png]

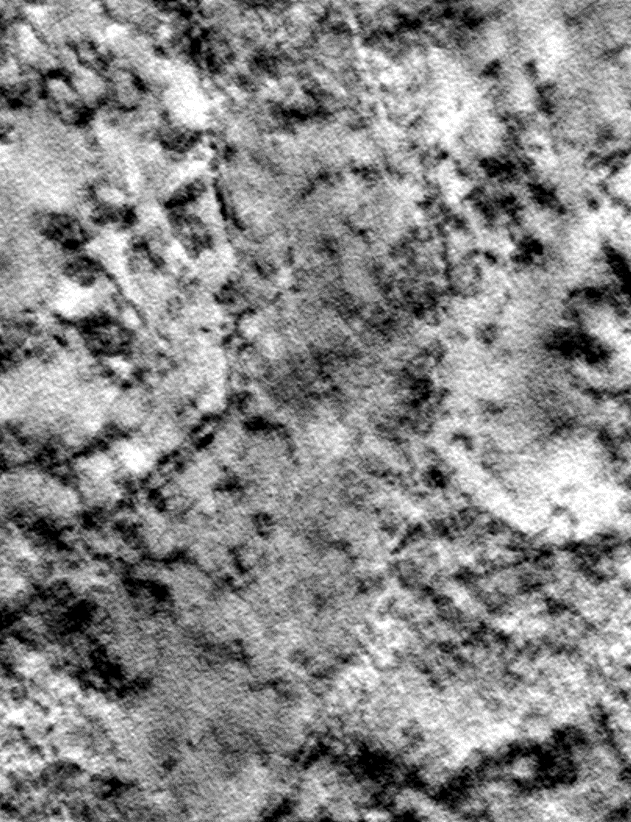

Supplement: Supplementary file 10 — Source data Fig. 3 [file 44319_2024_175_MOESM10_ESM.zip › Figure 3/3A/Fig3A_6.png]

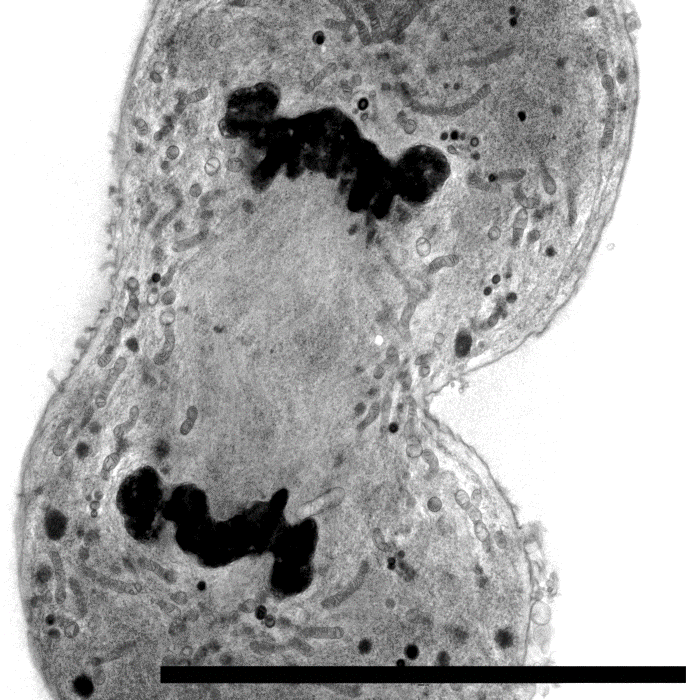

Supplement: Supplementary file 10 — Source data Fig. 3 [file 44319_2024_175_MOESM10_ESM.zip › Figure 3/3D/Fig3D_1.png]

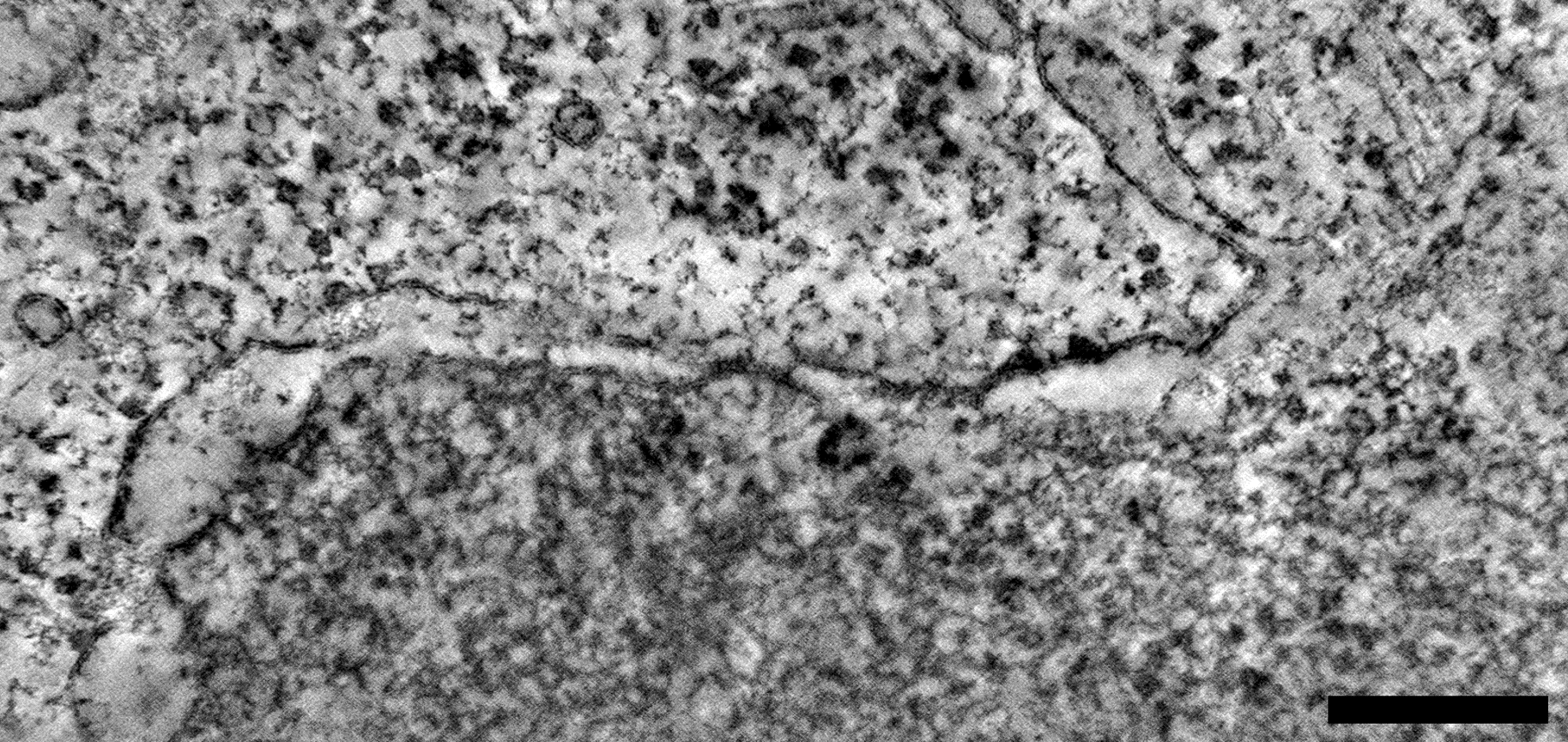

Supplement: Supplementary file 10 — Source data Fig. 3 [file 44319_2024_175_MOESM10_ESM.zip › Figure 3/3D/Fig3D_2.png]

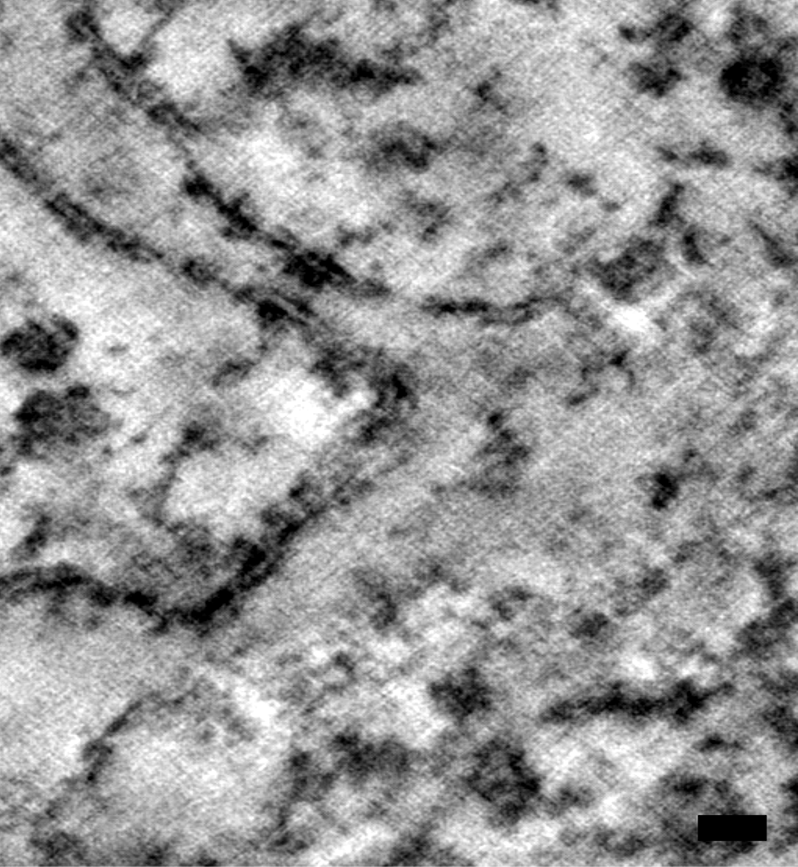

Supplement: Supplementary file 10 — Source data Fig. 3 [file 44319_2024_175_MOESM10_ESM.zip › Figure 3/3D/Fig3D_3.png]

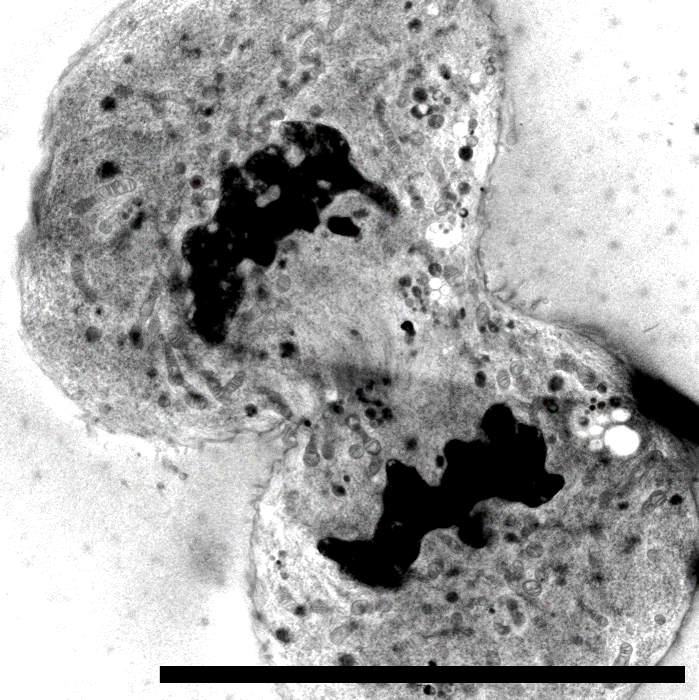

Supplement: Supplementary file 10 — Source data Fig. 3 [file 44319_2024_175_MOESM10_ESM.zip › Figure 3/3D/Fig3D_4.png]

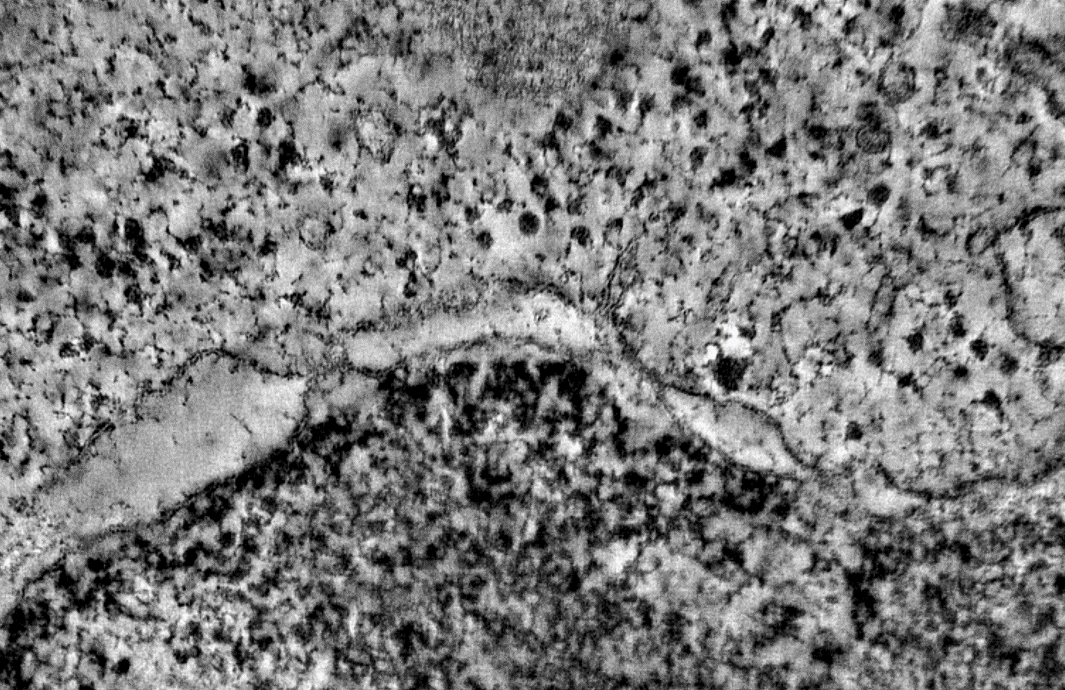

Supplement: Supplementary file 10 — Source data Fig. 3 [file 44319_2024_175_MOESM10_ESM.zip › Figure 3/3D/Fig3D_5.png]

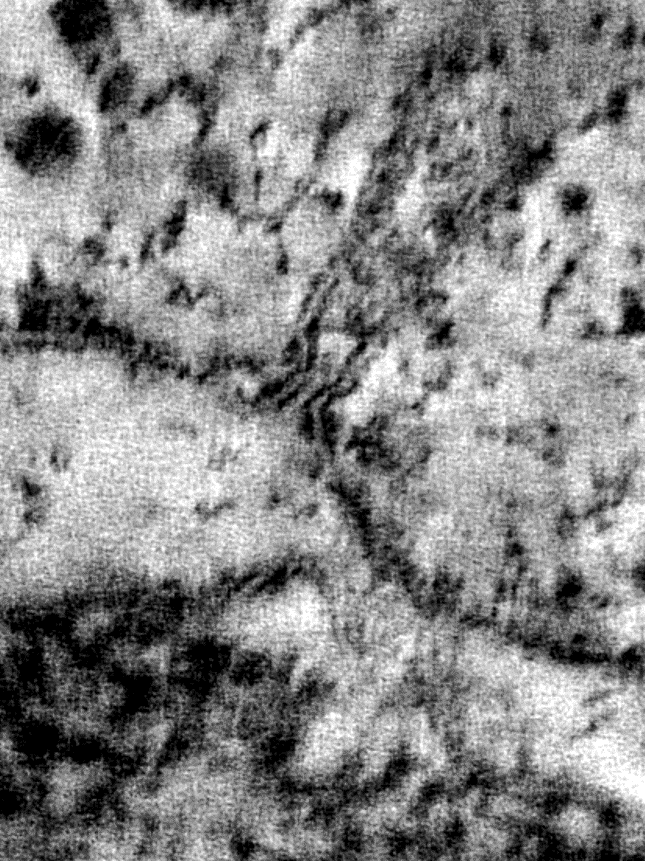

Supplement: Supplementary file 10 — Source data Fig. 3 [file 44319_2024_175_MOESM10_ESM.zip › Figure 3/3D/Fig3D_6.png]

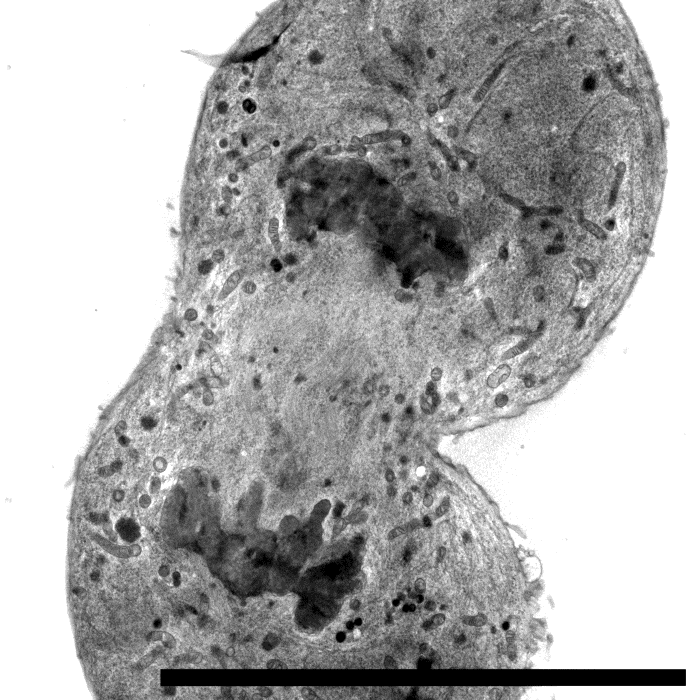

Supplement: Supplementary file 11 — Source data Fig. 4 [file 44319_2024_175_MOESM11_ESM.zip › Figure 4/4A/Fig4A_1.png]

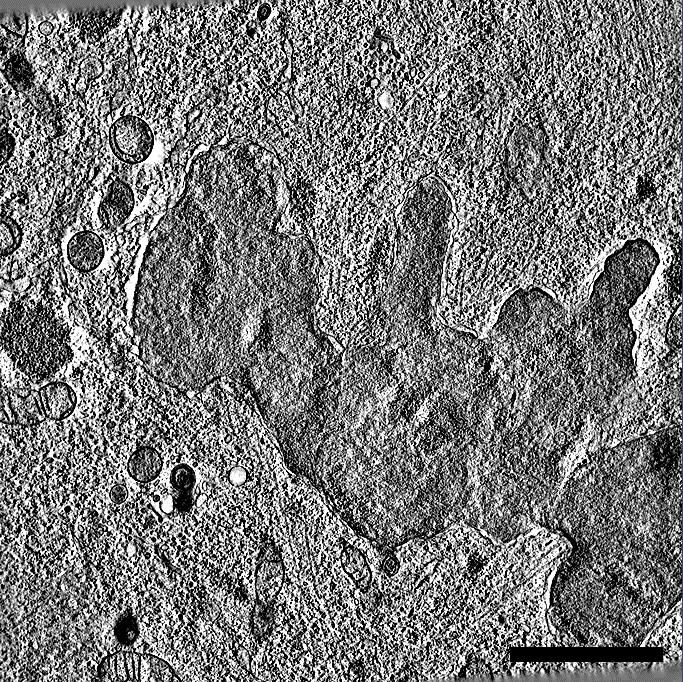

Supplement: Supplementary file 11 — Source data Fig. 4 [file 44319_2024_175_MOESM11_ESM.zip › Figure 4/4A/Fig4A_2.png]

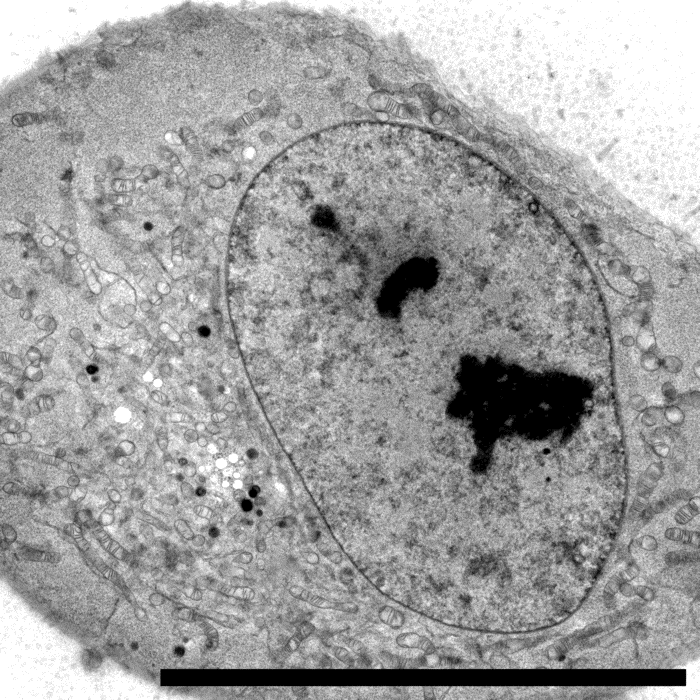

Supplement: Supplementary file 11 — Source data Fig. 4 [file 44319_2024_175_MOESM11_ESM.zip › Figure 4/4B/Fig4B_1.png]

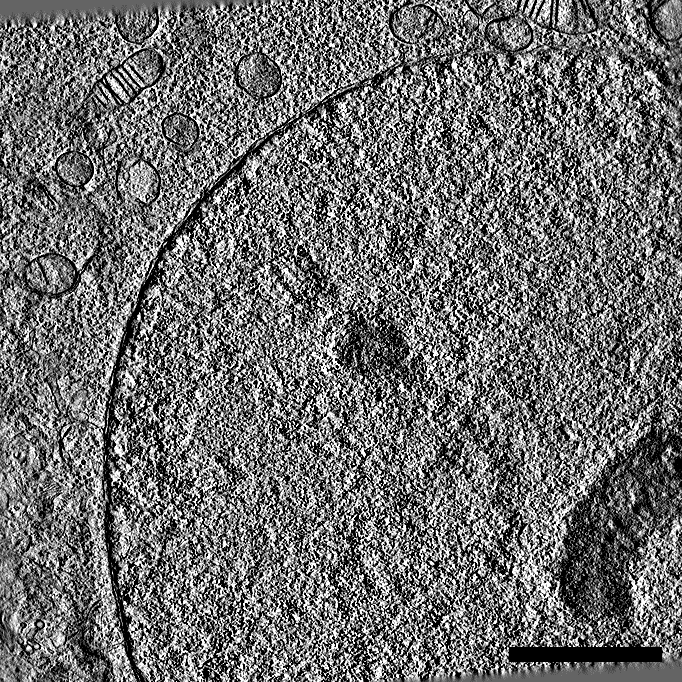

Supplement: Supplementary file 11 — Source data Fig. 4 [file 44319_2024_175_MOESM11_ESM.zip › Figure 4/4B/Fig4B_2.png]

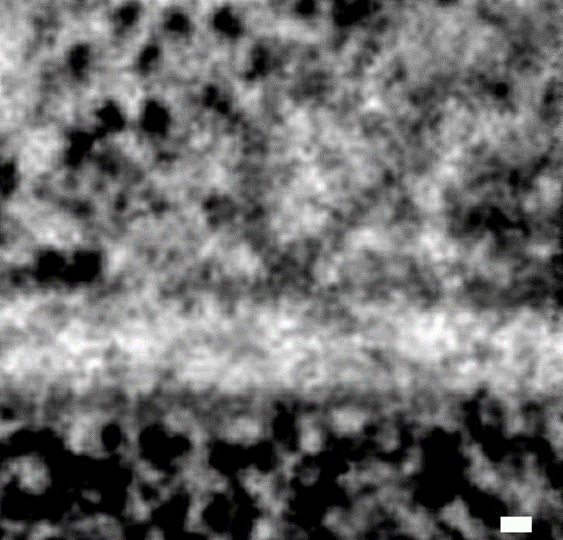

Supplement: Supplementary file 12 — Source data Fig. 5 [file 44319_2024_175_MOESM12_ESM.zip › Figure 5/5A/Fig5A_1.png]

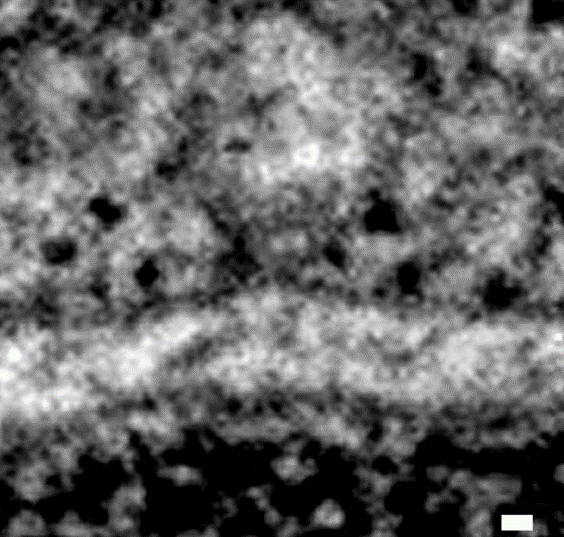

Supplement: Supplementary file 12 — Source data Fig. 5 [file 44319_2024_175_MOESM12_ESM.zip › Figure 5/5A/Fig5A_2.png]

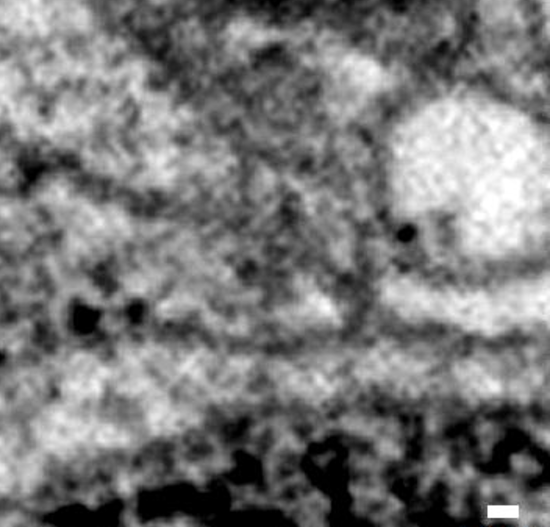

Supplement: Supplementary file 12 — Source data Fig. 5 [file 44319_2024_175_MOESM12_ESM.zip › Figure 5/5A/Fig5A_3.png]

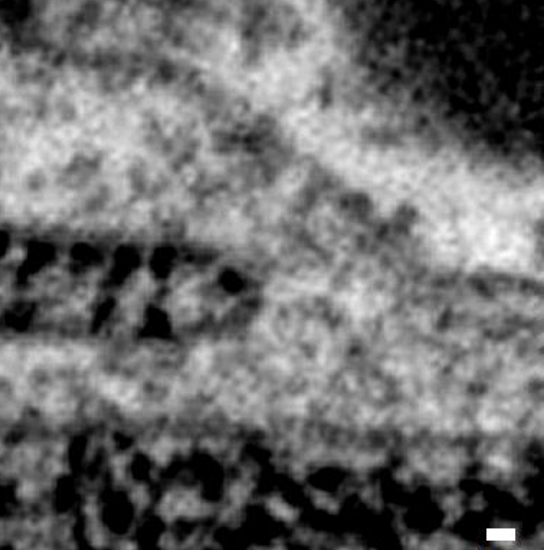

Supplement: Supplementary file 12 — Source data Fig. 5 [file 44319_2024_175_MOESM12_ESM.zip › Figure 5/5A/Fig5A_4.png]

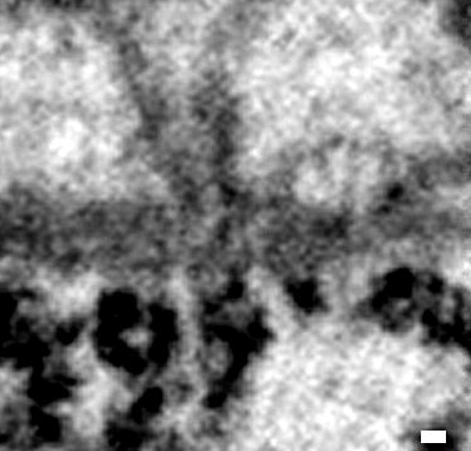

Supplement: Supplementary file 12 — Source data Fig. 5 [file 44319_2024_175_MOESM12_ESM.zip › Figure 5/5B/Fig5B_1.png]

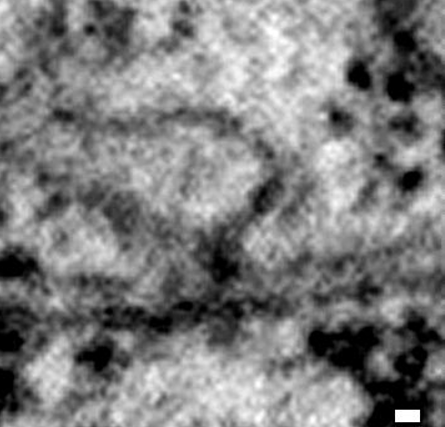

Supplement: Supplementary file 12 — Source data Fig. 5 [file 44319_2024_175_MOESM12_ESM.zip › Figure 5/5B/Fig5B_2.png]

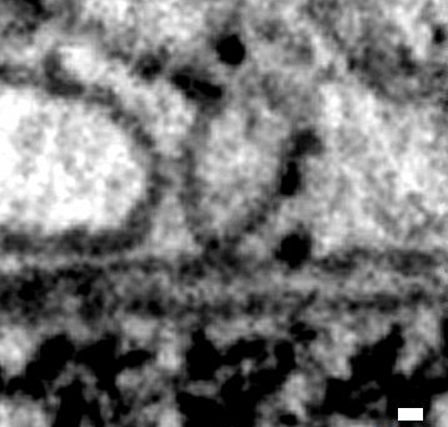

Supplement: Supplementary file 12 — Source data Fig. 5 [file 44319_2024_175_MOESM12_ESM.zip › Figure 5/5B/Fig5B_3.png]

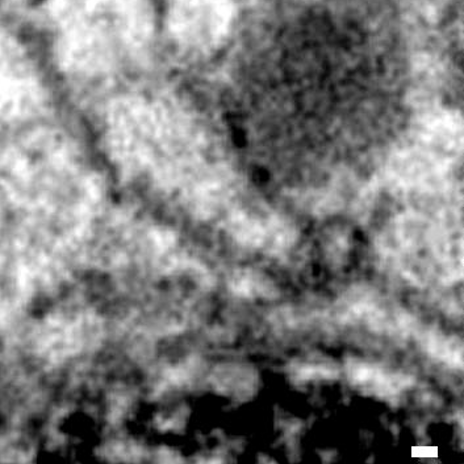

Supplement: Supplementary file 12 — Source data Fig. 5 [file 44319_2024_175_MOESM12_ESM.zip › Figure 5/5C/Fig5C_1.png]

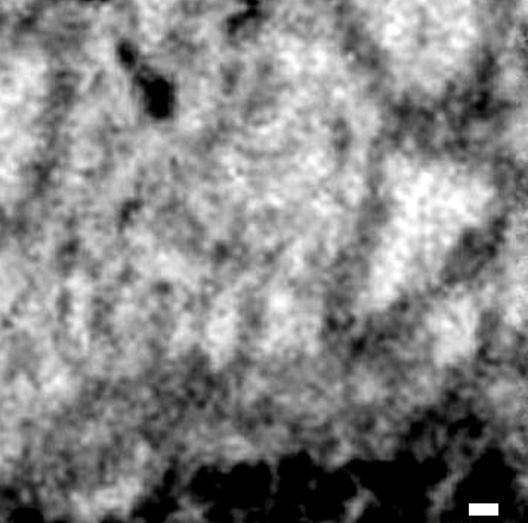

Supplement: Supplementary file 12 — Source data Fig. 5 [file 44319_2024_175_MOESM12_ESM.zip › Figure 5/5C/Fig5C_2.png]

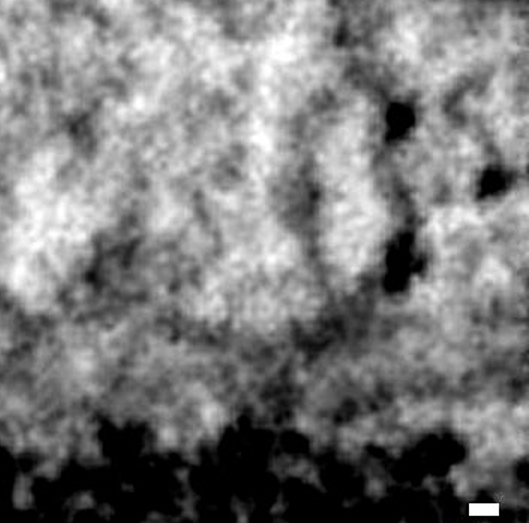

Supplement: Supplementary file 12 — Source data Fig. 5 [file 44319_2024_175_MOESM12_ESM.zip › Figure 5/5C/Fig5C_3.png]

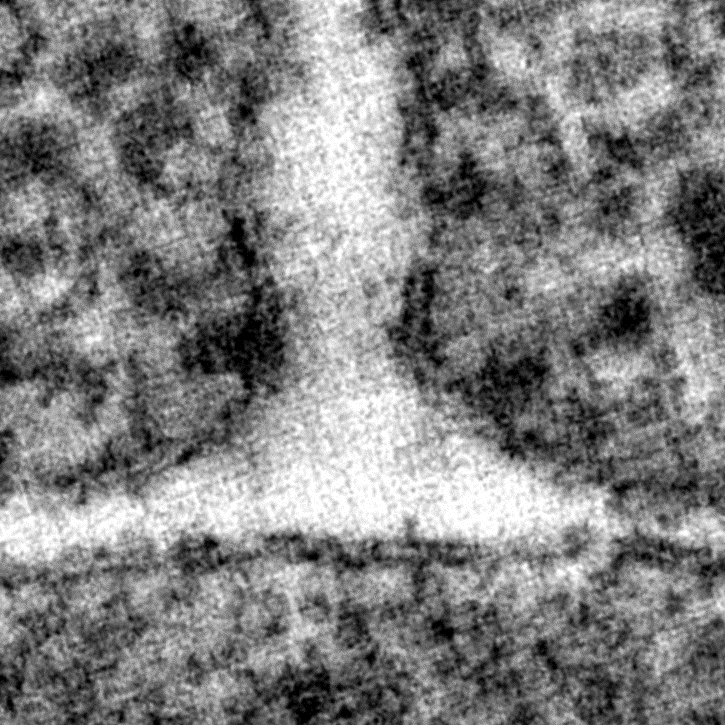

Supplement: Supplementary file 12 — Source data Fig. 5 [file 44319_2024_175_MOESM12_ESM.zip › Figure 5/5F/Fig5F_1.png]

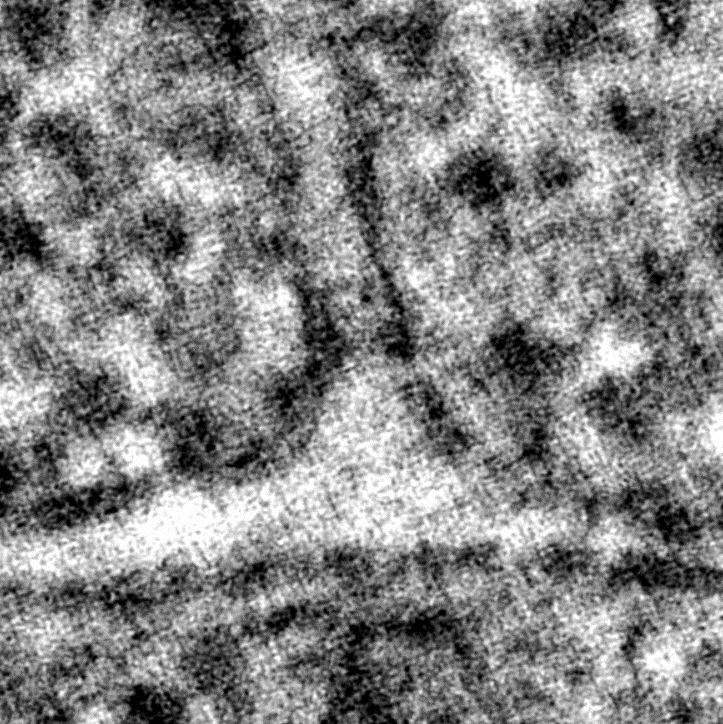

Supplement: Supplementary file 12 — Source data Fig. 5 [file 44319_2024_175_MOESM12_ESM.zip › Figure 5/5F/Fig5F_2.png]

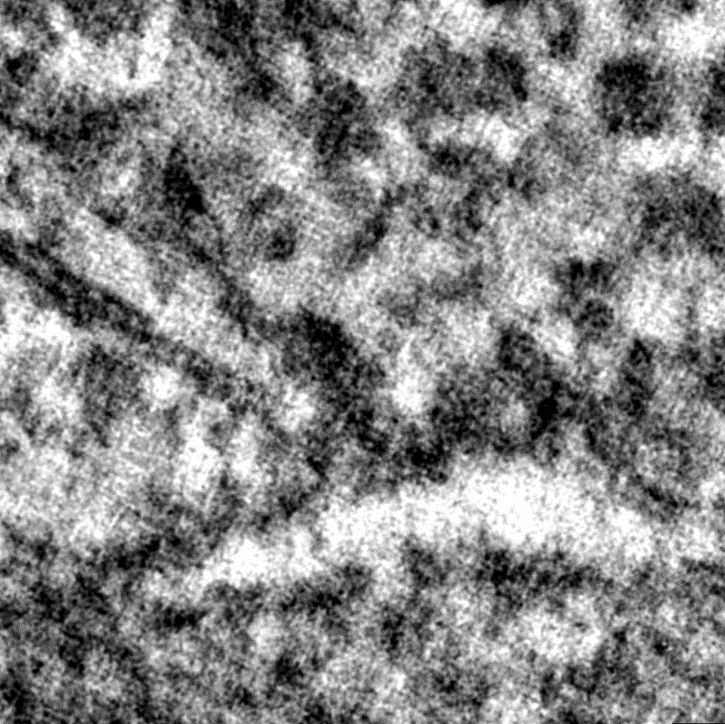

Supplement: Supplementary file 12 — Source data Fig. 5 [file 44319_2024_175_MOESM12_ESM.zip › Figure 5/5F/Fig5F_3.png]

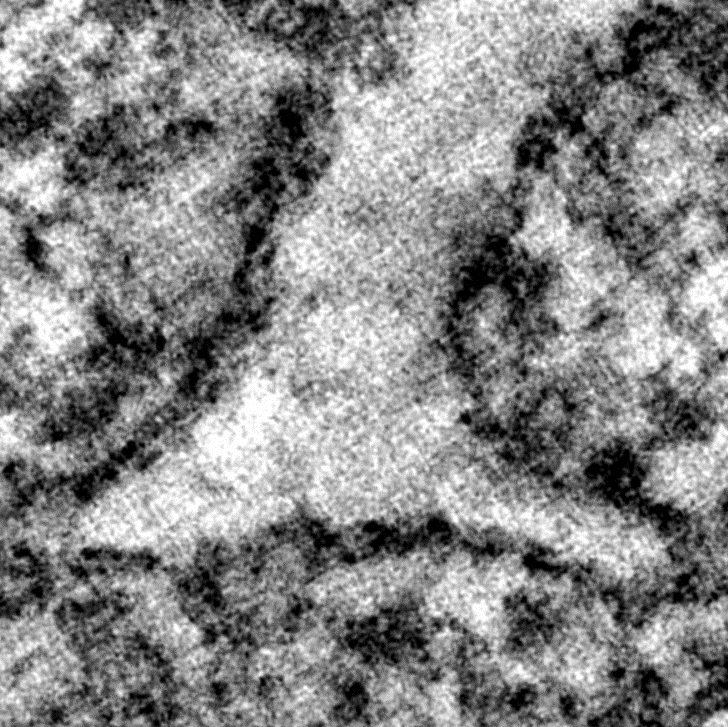

Supplement: Supplementary file 12 — Source data Fig. 5 [file 44319_2024_175_MOESM12_ESM.zip › Figure 5/5F/Fig5F_4.png]
