# Supplementary material for: The endoplasmic reticulum connects to the nucleus by constricted junctions that mature after mitosis
Source: EMBO Rep. 2024 Jun 14;25(7):18. doi: 10.1038/s44319-024-00175-w (PMC11239909; doi:10.1038/s44319-024-00175-w)
Supplement: Supplementary file 13 — Expanded View Figures [file 44319_2024_175_MOESM13_ESM.pdf]

Expanded View Figures

Figure EV1. Additional analyses of ER and NE morphology around the junctions in interphase.

(**A**) Additional examples of ER–NE junctions and different types of ER–NE contact sites in interphase. Electron tomographic slices at the sagittal plane of ER–NE junctions with and without lumen, ER–NE 'contact sites' where the ER membrane is juxtaposed to the ONM but their membranes are not fused, and where the ER is connected to the ONM via filamentous-like densities. For each junction, the left image shows raw EM data; the right one, the EM data on which the ER and the NE are coloured in orange and green, respectively. The filaments are indicated with magenta arrowheads. Scale bars: 20 nm. ER: endoplasmic reticulum, NE: nuclear envelope. See also Movie EV3. (**B**) Membrane profiles of the side views of ER–NE junctions with and without lumen found in interphase cells. To distinguish individual junctions, the profiles are coloured differently for each junction. (**C**) Morphology of the ER at 100 nm away from the junction base that connects to the NE and to the flat sheet of the ER that were analysed in this study. (i) The 3D meshes of the junctions in which the ER pieces connecting to the flat membrane are tubule-like, sheet-like, and with other morphology. Their 90°-rotated views are shown below. White arrowheads point to regions 100 nm away from the junction base. Note that in tubules the width stays similar after 90° rotation while in sheets the width gets much thinner. Scale bar: 100 nm. (ii) The proportion of each morphological type of the ER connecting to the NE (left) and the flat sheet of the ER (right). (**D**) Local NE dilation below ER–NE junctions. (i) A scheme depicting that the width of the perinuclear space was measured below ER–NE junctions and 200–500 nm away. (ii) Quantification of the NE width below ER–NE junctions (with and without lumen, $n = 10$ and 9, respectively) and 200–500 nm away from each junction ($n = 20$ and 18, respectively) from 9 cells from a single experiment. *p-value < 0.05; two-tailed Mann–Whitney test. n.s.: not significant (p-value > 0.5). The median is depicted as a horizontal line. (**E**) Distance from the ER–ER junctions that were analysed in Fig. 2F–I to the outer nuclear membrane (ONM). $n = 14$ junctions from 4 cells from a single experiment. The median is depicted as a horizontal line. (**F**) An ER piece forming simultaneously a constricted junction to the NE and a wide junction to an ER sheet. The left image shows raw EM data; the middle one, the EM data on which the ER and the NE are coloured in orange and green, respectively. The right image shows the 3D mesh of the ER and the NE. The ER–ER and ER–NE junctions are indicated by white arrowheads. Scale bar: 200 nm. C: cytoplasm, N: nucleus. See also Movie EV4.

© The Author(s)

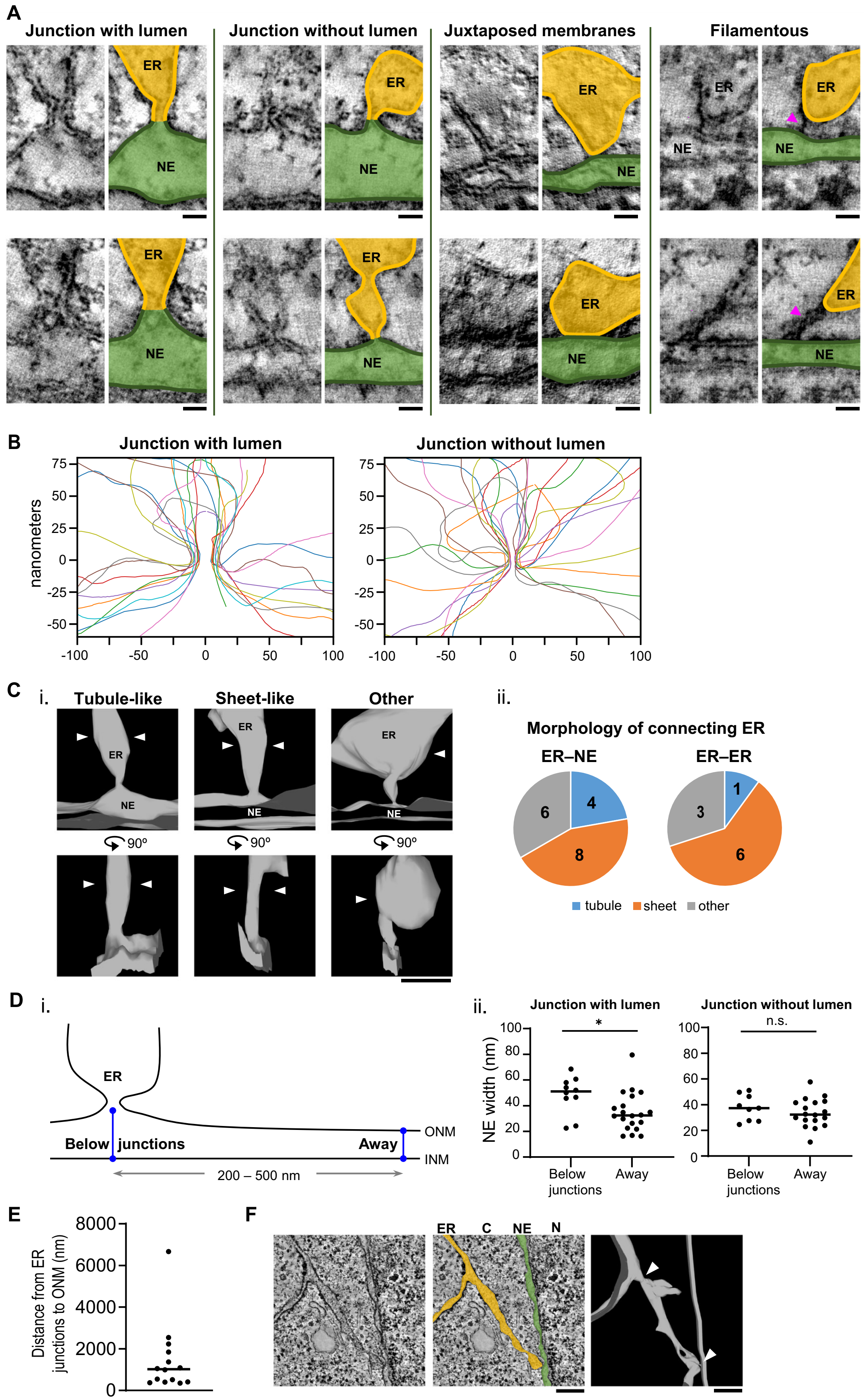
A
Junction with lumen
Junction without lumen
Juxtaposed membranes
Filamentous
ER
NE
B
Junction with lumen
Junction without lumen
nanometers
C
i.
Tubule-like
Sheet-like
Other
90°
ii.
Morphology of connecting ER
ER–NE
ER–ER
tubule
sheet
other
D
i.
ER
Below junctions
Away
ONM
INM
200 – 500 nm
ii.
Junction with lumen
Junction without lumen
NE width (nm)
*
n.s.
Below junctions
Away
E
Distance from ER junctions to ONM (nm)
F
ER
C
NE
N

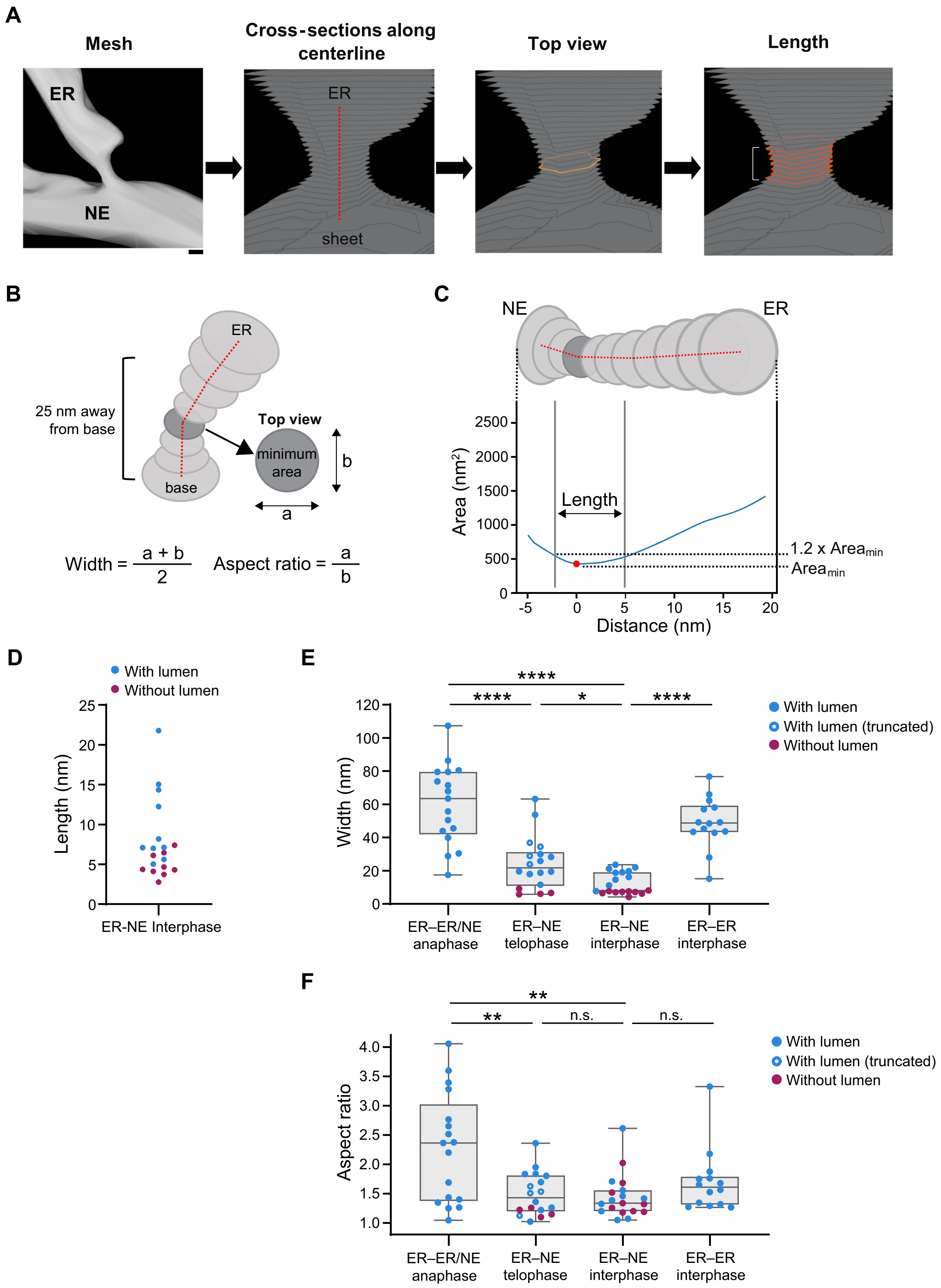
A
Mesh
Cross-sections along centerline
Top view
Length
ER
NE
ER
sheet
B
ER
25 nm away from base
Top view
minimum area
base
a
b
Width = (a + b)/2
Aspect ratio = a/b
C
NE
ER
Area (nm²)
Length
1.2 x Area min
Area min
Distance (nm)
D
With lumen
Without lumen
Length (nm)
ER-NE Interphase
E
With lumen
With lumen (truncated)
Without lumen
Width (nm)
ER–ER/NE anaphase
ER–NE telophase
ER–NE interphase
ER–ER interphase
F
Aspect ratio
n.s.

Figure EV2. 3D-ultrastructural analysis of junctions.

(**A**) To quantify the 3D ultrastructure of the junctions, cross-sections were obtained at regularly-spaced intervals along a centreline of the 3D meshes. The cross-sections allowed identifying the junction top view and length. Scale bar: 20 nm. (**B**) The cross-section with the minimum surface area was selected up to 25 nm from the base for making a top-view profile. The schemes represent how to determine the width and the aspect ratio of junctions. (**C**) A plot showing the cross-sectional area along the junction axis from the NE to the ER. The curve is centred at the cross-section with the minimum surface area. The distance between the two cross-sections with a surface area 1.2 times larger than the minimum one was defined as the length of the junctions. (**D**) Length of ER–NE junctions in interphase ($n = 19$ from 9 cells from a single experiment). The plots for the junctions with and without lumen are colour-coded in blue and magenta, respectively. (**E**) Combined plots of the width of ER–ER/NE junctions in anaphase, ER–NE junctions in telophase and interphase, as well as the width of ER–ER junctions in interphase. $n = 17$, 18, 19, and 14 for ER–NE anaphase, telophase, interphase, and ER–ER interphase, from 3, 2, 9, and 4 cells, respectively, from a single experiment. Dots with a white filling indicate the 4 truncated top view profiles, whose width is underestimated. **p*-value < 0.05, *****p*-value < 0.0001; two-tailed Mann–Whitney test. Centre line, median; box limits, upper and lower quartiles; whiskers, min and max. (**F**) Combined plots of the aspect ratio of the junctions. The plots are shown as in (**E**). $n = 17$, 18, 19 and 14 for ER–NE anaphase, telophase, interphase, and ER–ER interphase, from 3, 2, 9, and 4 cells, respectively, from a single experiment. ***p*-value < 0.01; two-tailed Mann–Whitney test. n.s.: not significant (*p*-value > 0.5).

© The Author(s)

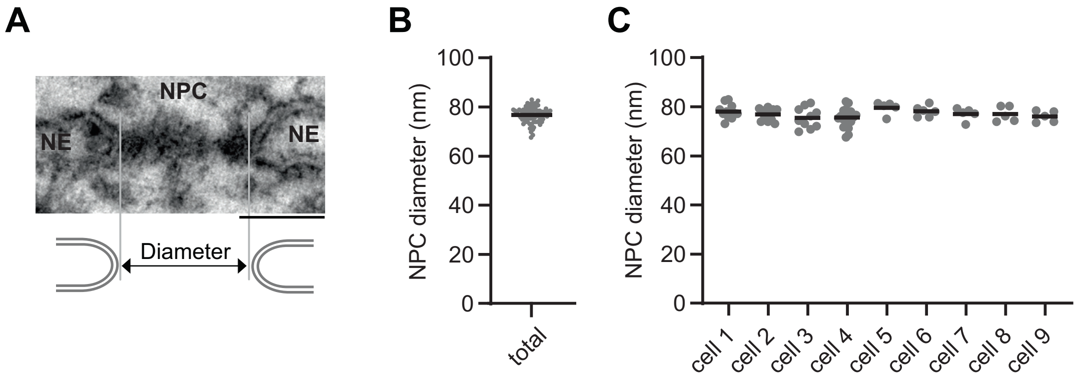

Figure EV3. Quantification of nuclear pore diameter to assess the degree of shrinkage in the EM tomograms.

(**A**) An electron tomographic slice of a nuclear pore in interphase. Average intensity projection of 20 z-slices (corresponding to 9 nm). Scale bar: 50 nm. Inner nuclear pore diameter was measured as indicated by a bidirectional arrow in the bottom panel. NPC: nuclear pore complex, NE: nuclear envelope. (**B**) Nuclear pore diameter in the EM tomograms in which we inspected ER–NE and ER–ER junctions. The average diameter was 77 nm ± 3.2 nm (mean ± S.D., 80 pores in 9 cells from a single experiment). Since the nuclear pore diameter was 92 nm in cryo-EM tomograms of interphase HeLa cells that were vitrified by plunge freezing (Mosalaganti et al, 2022), the shrinkage of the specimen in our EM tomograms is estimated to be 17%. (**C**) Comparison of nuclear pore diameter among the interphase cells analysed by EM tomography in this study. The diameter was comparable between different cells ($n = 12$, 12, 10, 20, 5, 6, 5, 5, 5 nuclear pores in 9 cells from a single experiment), indicating that the shrinkage occurred to a similar degree in the EM tomograms of cells that we inspected.

 © The Author(s)

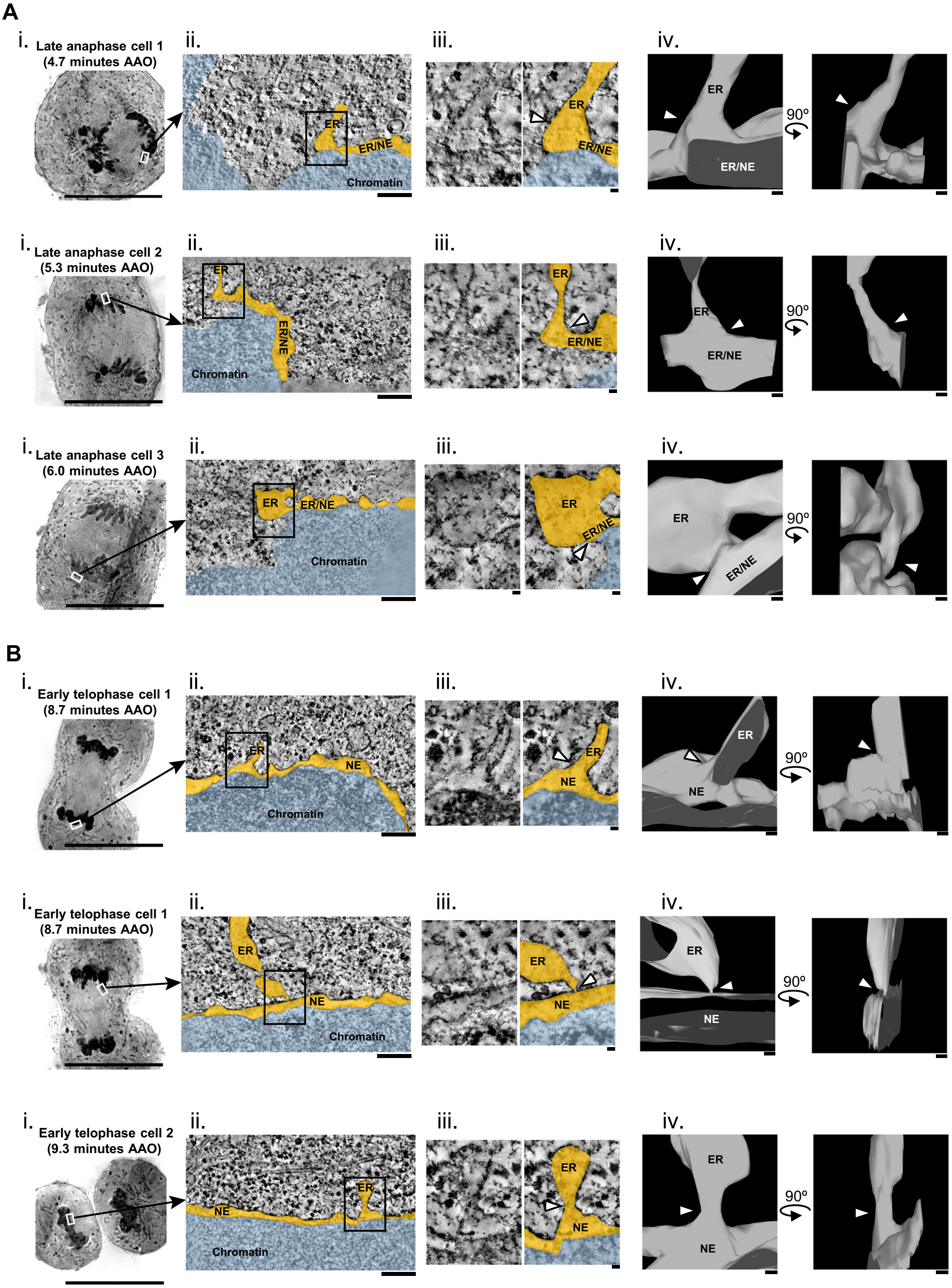
A
i. Late anaphase cell 1
(4.7 minutes AAO)
ii.
ER
ER/NE
Chromatin
iii.
ER
ER/NE
iv.
ER
ER/NE
90º
i. Late anaphase cell 2
(5.3 minutes AAO)
ER
ER/NE
Chromatin
ER
ER/NE
ER
ER/NE
90º
i. Late anaphase cell 3
(6.0 minutes AAO)
ER
ER/NE
Chromatin
ER
ER/NE
ER
ER/NE
90º
B
i. Early telophase cell 1
(8.7 minutes AAO)
ER
NE
Chromatin
ER
NE
ER
NE
90º
i. Early telophase cell 1
(8.7 minutes AAO)
ER
NE
Chromatin
ER
NE
ER
NE
90º
i. Early telophase cell 2
(9.3 minutes AAO)
ER
NE
Chromatin
ER
NE
ER
NE
90º

◀ **Figure EV4. Additional examples of junctions in late anaphase and in early telophase.**

(**A**) Additional example images of ER junctions contacting the chromatin in late anaphase cells. (i) 2D EM micrographs of three different late anaphase cells. AAO: After Anaphase Onset. (ii) Tomographic slices of cells in late anaphase showing that the ER (orange) starts to contact the chromatin (blue), but the NE is still indistinguishable. (iii) Enlargement of the regions indicated in (ii). For each junction, the left image shows raw EM data; the right one, the EM data on which the ER and the chromatin are coloured in orange and blue, respectively. (iv) 3D meshes of the junctions and their 90°-rotated views. Junctions are indicated by white arrowheads in (iii) and (iv). (**B**) Additional example images of ER–NE junctions in early telophase. Images are displayed in the same way as in (**A**). Data Information: Scale bars for (i): 20 µm; Scale bars for (ii): 200 nm; Scale bars for (iii, iv): 20 nm.

 © The Author(s)

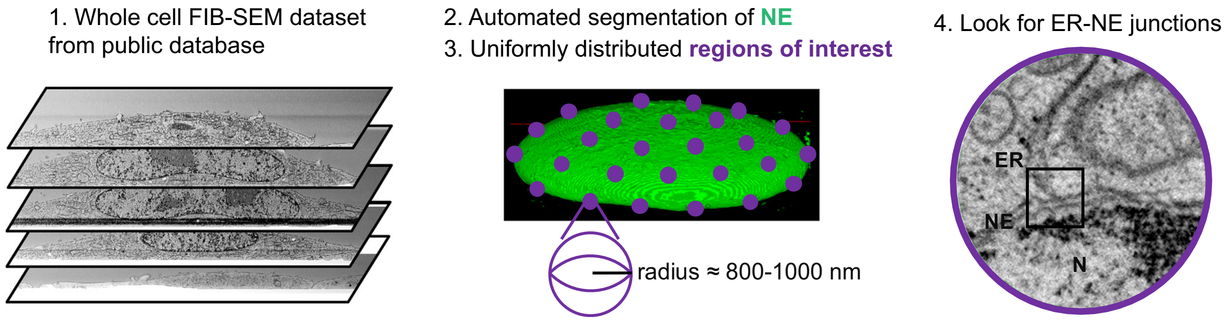

Figure EV5. Stereology-based approach to find ER–NE junctions in FIB-SEM datasets of entire cells.

The datasets were downloaded from OpenOrganelle (Heinrich et al, 2021: Data ref: Heinrich et al, 2021; Xu et al, 2021: Data ref: Xu et al, 2021). On the surface of automatically-segmented nuclei, the regions of interest were uniformly sampled with a radius of 800–1000 nm. In these regions, potential ER–NE junctions were searched manually.
